# Targeting Cell Entry of Enveloped Viruses as an Antiviral Strategy

**DOI:** 10.3390/molecules16010221

**Published:** 2010-12-30

**Authors:** Elodie Teissier, François Penin, Eve-Isabelle Pécheur

**Affiliations:** Institut de Biologie et Chimie des Protéines, UMR 5086, Université de Lyon, IFR 128 BioSciences Gerland-Lyon Sud, 69367 Lyon, France; E-Mails: e.teissier@ibcp.fr (E.T.); f.penin@ibcp.fr (F.P.)

**Keywords:** inhibitors, viral entry, enveloped virus

## Abstract

The entry of enveloped viruses into their host cells involves several successive steps, each one being amenable to therapeutic intervention. Entry inhibitors act by targeting viral and/or cellular components, through either the inhibition of protein-protein interactions within the viral envelope proteins or between viral proteins and host cell receptors, or through the inhibition of protein-lipid interactions. Interestingly, inhibitors that concentrate into/onto the membrane in order to target a protein involved in the entry process, such as arbidol or peptide inhibitors of the human immunodeficiency virus (HIV), could allow the use of doses compatible with therapeutic requirements. The efficacy of these drugs validates entry as a point of intervention in viral life cycles. Strategies based upon small molecule antiviral agents, peptides, proteins or nucleic acids, would most likely prove efficient in multidrug combinations, in order to inhibit several steps of virus life cycle and prevent disease progression.

## Introduction

**Figure 1:**
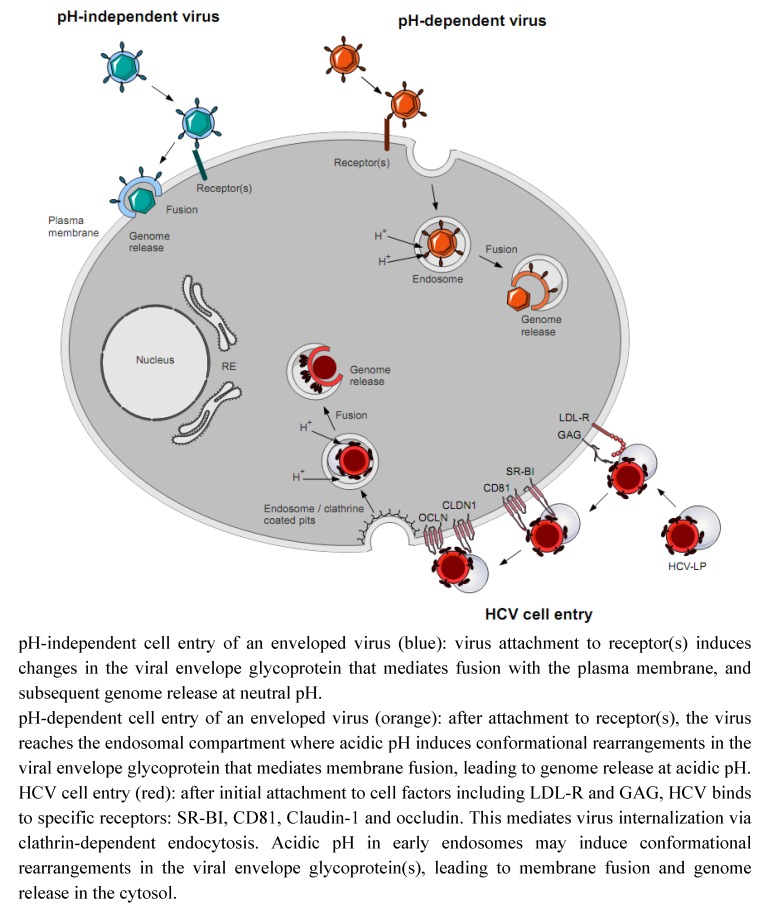
Schematic model of cell entry of enveloped viruses.

Therapeutic inhibition of virus infection could involve several strategies and target various steps of virus life cycle such as cell entry, virus replication or the assembly and release of newly-formed virions. In this review we will focus on strategies aimed at inhibiting the cell entry step of enveloped viruses. Major efforts have been invested to find novel, specific and less toxic antiviral strategies. Targeting the entry of enveloped viruses is a very attractive strategy for therapeutic intervention since the site of action of the inhibitor is likely to be extracellular and therefore relatively accessible; this could also limit cell toxicity. Moreover one could expect the beneficial effects upstream to damages that would occur to the cell later in the viral life cycle. The penetration of an enveloped virus into its target cell is the first step of the viral replication cycle, and each stage could constitute a potential target for an inhibitor (Figure 1) [1].

Schematically, to enter the cell, enveloped viruses first attach to one or several plasma membrane receptors *via* their envelope proteins. In the case of a pH-independent entry, this initial binding leads to conformational changes of the envelope fusion protein, eventually exposing the fusion peptide. Insertion of this peptide into the plasma membrane allows fusion between viral and plasma membranes, and release of the virus genome into the cytoplasm. Membrane fusion of viruses displaying pH-dependent entry occurs within the acidic environment of endosomal compartments, where the pH induces the conformational changes of the fusion protein leading to the exposure of the fusion peptide.

All these stages are potentially amenable to therapeutic intervention. In this review, we will therefore discuss the various possible targets in the context of recently developed antiviral molecules. The knowledge and understanding of antiviral strategies studied or applied for enveloped viruses could lead to the development of new inhibitors.

## Three Classes of Viral Fusion Proteins

**Figure 2:**
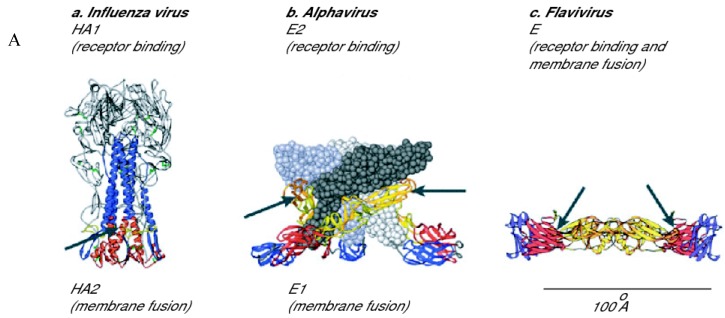

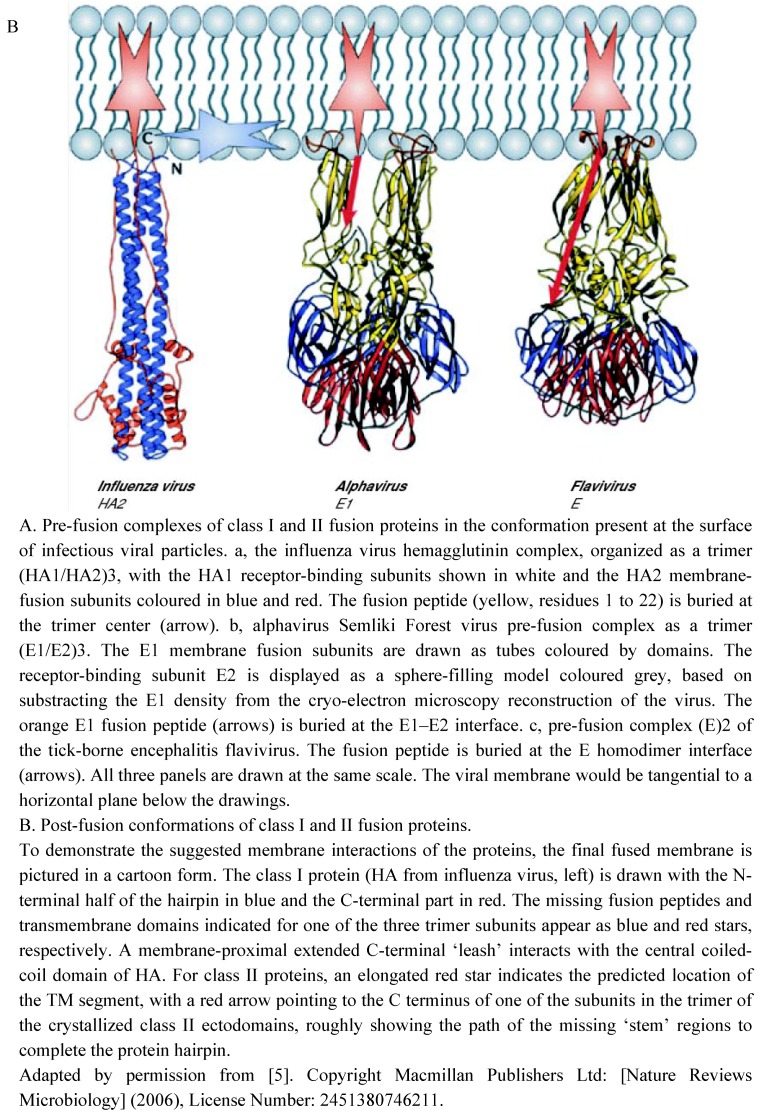
Conformation of enveloped virus proteins of classes I and II.

**Figure 3:**
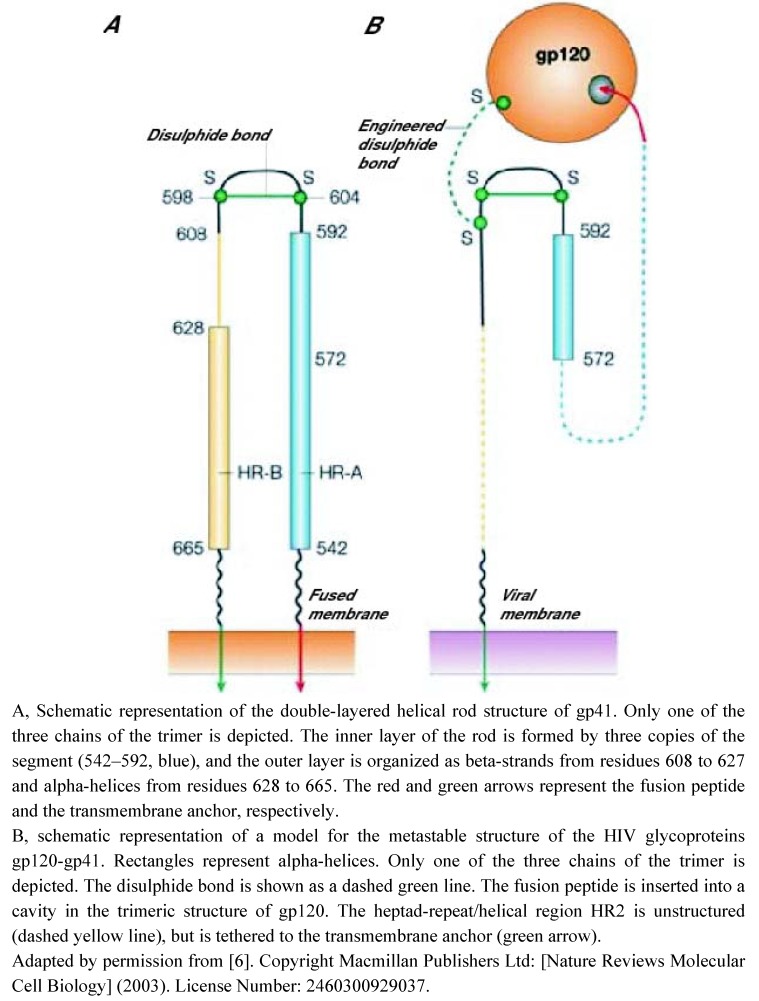
Schematic structure of HIV gp41 in pre- and post-fusion conformations.

Viral envelope proteins have two functions: the specific attachment to the cell and membrane fusion. These two functions could involve one or more proteins. Viral fusion proteins are defined as three classes, mainly depending on structural features [2,3,4,5]. Fusion proteins of these three classes display different architectures but they adopt during fusion a similar overall ‘hairpin’ conformation [5] (Figure 2 and Figure 3). However, for many virus families, structural information is still missing to identify and classify their membrane fusion proteins. 

Class I proteins mostly contain alpha-helical structures and the hydrophobic fusion peptide is located at the N-terminus, buried within the protein core. These proteins associate as homotrimers that project vertically from the viral membrane. This group includes proteins of several viral families as *Retroviridae* (Human Immunodeficiency Virus HIV, gp41), *Orthomyxoviridae* (Influenza virus, HA protein*), Paramyxoviridae* (Respiratory Syncytial Virus RSV, F protein), *Filoviridae* (Ebola, GP2 protein) and *Coronaviridae* (Coronavirus, S protein) [5].

Viruses with class I fusion proteins share a common mechanism of fusion, mediated by a glycoprotein that contains two hydrophobic heptad repeats in its extracellular domain, which are central to the complex conformational changes leading to fusion. The first heptad repeat (HR1 or HRN) is adjacent to the fusion peptide, while the second (HR2 or HRC) immediately precedes the transmembrane domain (Figure 2). 

Once fusion is triggered, these domains drastically rearrange to form highly structured and thermodynamically stable coiled-coils of three HR1 heptad repeats in a prefusion stalk conformation. This intermediate allows the fusion peptide to be exposed and eventually inserted into the membrane of the target cell. In the final stage of membrane fusion, the pre-hairpin spontaneously collapses into the post-fusion structure - a six-helical bundle (6HB), with the inner trimeric coiled coil formed by the HR1 onto which the HR2 folds (Figure 2). This results in membrane merging and formation of a stable fusion pore [7,8,9]. 

Concerning HIV, envelope proteins are arranged as two non covalently-linked glycoproteins, gp120 (function of attachment) and gp41 (function of fusion), forming trimeric spikes projecting from the lipid viral envelope [10] (Figure 3). To enter the cell, HIV specifically binds to the host protein receptor CD4 *via* its gp120 subunit [11,12]. CD4 binding induces a conformational change exposing an otherwise cryptic binding site on the gp120 molecule for one of the two major HIV coreceptors, CCR5 or CXCR4. Coreceptor binding triggers conformational changes in gp120 and gp41, resulting in the exposure of the fusion peptide, its insertion into the plasma membrane and the association of HR2 domains with the HR1 trimer, to form a “hairpin” structure forming the thermodynamically stable 6HB (Figure 3). This allows membrane fusion and the release of the genome of HIV directly in the cell cytoplasm. Interestingly, cholesterol-enriched lipid microdomains of the plasma membrane seem required for HIV cell entry [13].

Two viral glycoproteins are involved in cell infection by the respiratory syncytial virus (RSV): an attachment protein (G) and the fusion protein (F) [14,15]. These proteins bind to heparin and to cell surface glycoproteins allowing attachment. However this process remains unclear : the interaction of the G protein with its receptors is thought to initiate the conformational changes in F to form the HR1/HR2 6HB, thereby triggering membrane fusion at neutral pH at the plasma membrane.

The HA hemagglutinin of the influenza virus anchors the virus to its target cells by attachment to sialic acid-rich receptors. The virus then enters the cell by clathrin-dependent endocytosis [16] and, in the acidic environment of the endosomes, the low pH of the endosome activates the M2 ion channel to bring protons across the viral envelope, which results in the acidification of the viral interior. Then, HA trimers mediate the fusion of viral and endosomal membranes. Thereafter acidification of the viral nucleocapsid allows genome release into the host cell cytoplasm [17,18].

**Figure 4:**
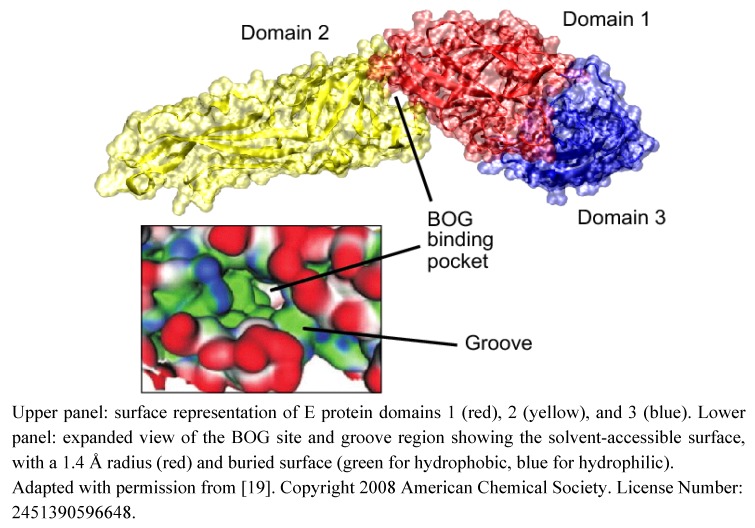
Monomeric dengue E protein showing the targeted beta-octyl glucoside (BOG) site.

Conversely, class II fusion proteins mainly consist of beta-sheets, and the fusion peptide is located in internal loops (Figure 2). Unlike class I proteins, which remain trimeric, their conformational rearrangements involve a change in their oligomeric state, from pre-fusion dimers lying onto the virion surface to post-fusion trimers that project vertically. Class II fusion proteins are the hallmark of viruses belonging to the *Flaviviridae* family, notably in the Flavivirus genus with the E fusion protein of the Dengue virus (DENV), Yellow Fever virus (YFV) or West Nile virus (WNV), and of viruses belonging to the *Togaviridae* family, in particular the E1 protein of the alphavirus Semliki Forest (SFV) [5]. Class II fusion proteins are characterized by three globular domains composed almost entirely of beta-sheets (Figure 4).

A hinge region is located between domains I and II. This flexible zone allows different angles between the two domains in the pre- and post-fusion conformations of fusion protein. The crystal structure of the Dengue virus E protein revealed a pocket located within this hinge region, and named the β−OG pocket. This site could accomodate a single β−OG molecule (Figure 4) [20]. This hydrophobic pocket is important during the conformational changes promoting fusion. Thus, blocking this pocket or impairing flexibility around the hinge region could impede the fusion reaction. Envelope proteins of class II are organized as homo- or hetero-dimers lying flat at the surface of the virion. Both Alphaviruses and Flaviviruses infect cells by receptor-mediated endocytic uptake and a low pH-triggered membrane fusion reaction. Before fusion, the E protein of Flaviviruses and the E1-E2 proteins of Alphaviruses form metastable dimers on the viral surface [4,5,21]. At the onset of fusion, interactions within the dimer are disrupted, releasing a monomeric fusion protein and leading to the exposure of fusion loops which insert into the target membrane. Then, the fusion protein forms the homotrimer that is required for fusion. Hinge rotation and dramatic rearrangement of domain III lead to the formation of a hairpin, with the fusion loops and the transmembrane domains at the same end of a rod-like trimer (Figure 2). This results in the release of viral genome into the cell.

Since HCV is a member of the *Flaviviridae* family, its fusion protein(s) is (are) assumed to belong to class II. The virion envelope bears two viral glycoproteins, E1 and E2, of currently unknown 3D-structure. This renders difficult the study of the HCV fusion mechanism at the molecular level. However, a model of the tertiary organization of E2 was recently proposed, based upon the identification of disulfide bonds and delineating a candidate fusion loop [22]. Furthermore, the study of HCV cell entry has been largely hampered until the development of two functional models, namely HCV pseudotyped particles (HCVpp) in 2003 and HCV grown in cell cultures (HCVcc) in 2005 [23,25]. To enter hepatocytes, its main target cells for productive infection, HCV binds a set of receptors and coreceptors including the low density lipoprotein receptor (LDL-R), glycosaminoglycans (GAG), scavenger receptor class B type I (SR-BI), the tetraspanin CD81, claudin-1 and occludin (Figure 1) [25,26,27,28,29,30]. Internalization is most likely achieved by clathrin-dependent endocytosis followed by low pH-dependent membrane fusion within the endosomes, expected to induce conformational changes in HCV E1/E2 and the release of HCV genome into the cell [31,32].

Fusion proteins of the third viral class (class III) display a combination of alpha-helical and beta-structures [33]. Like class I proteins, they form trimers in their pre-fusion state and contain a central alpha-helical coiled-coil. However, their fusion region more closely resemble that of class II proteins, since their fusion peptides form loops exposed at the tips of extended beta-strands. This last group includes fusion proteins from the *Rhabdoviridae* (G protein of the Vesicular Stomatitis Virus), the *Herpesviridae* (gB of the Herpes Simplex virus-1) and the *Baculoviridae* (gp64 of the Baculovirus) [33]. We will now focus on several viral entry inhibitors, and most promising molecules or strategies that are already in clinics are summarized in Table 1.

## Inhibitors Preventing Virus Attachment to Target Cells

### Antibodies

The development of neutralizing monoclonal antibodies (mAb) is a very promising antiviral strategy, however at present only palivizumab has demonstrated clinical efficacy. It was licensed in 1998 by the US Food and Drug Administration (FDA), and subsequently by the different world drug regulatory agencies for the prevention of severe RSV infections in high-risk children [34,35]. Motavizumab (MEDI-524) (Table 1) is a novel recombinant humanized IgG1 mAb developed from palivizumab. Motavizumab possesses enhanced anti-RSV neutralizing activity and is 18-fold more potent than palivizumab. A phase III study for the prevention of RSV infections in high-risk children has recently finished, and results indicated a superior efficacy of motavizumab compared to palivizumab in the population of patients with a history of prematurity [34]. The precise molecular mechanisms of action of these mAb are unknown; however they bind the RSV F protein [36]. A recent study demonstrated that they did not inhibit virus attachment or F protein interaction with the cell membrane, nor virus budding; however, these antibodies did inhibit virus transcription and cell-to-cell fusion [36]. This suggests that they could inhibit conformational changes in the F protein and thereby block fusion.

**Table 1:** Main molecules or strategies already in clinics or exhibiting most promising antiviral activities.

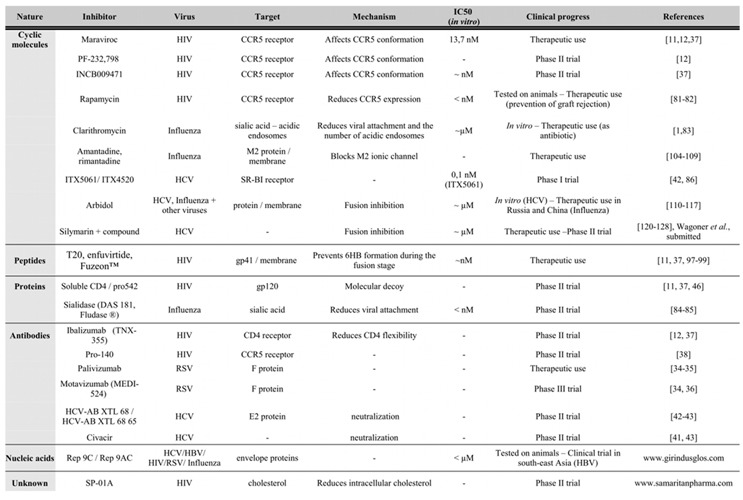

For HIV, several mAb are currently being investigated. Ibalizumab (TNX-355) (Table 1) is a humanized IgG4 mAb that binds to the second domain of the CD4 receptor [12,37]. It does not prevent gp120 binding to CD4, but is thought to decrease the flexibility of CD4, thereby hindering access of CD4-bound gp120 to CCR5 and CXCR4. Phase I studies of ibalizumab showed promising activity but resistance emerged after 9 weeks of administration. A phase II study of ibalizumab showed that this mAb in combination with an antiretroviral regimen significantly reduced plasma HIV-1 RNA compared to the background regimen alone. Another interesting mAb blocking HIV cell entry is Pro-140 that targets the CCR5 receptor; its efficacy has been evaluated in phase II clinical trials and it has been granted in 2006 fast-track status by the FDA [38]. In this study, subcutaneously-administered Pro-140 showed potent antiretroviral activity and the treatment was generally well tolerated. Pro-140 is currently in phase II clinical testing (www.progenics.com).

Concerning HCV, some antibodies directed against CD81 were studied *in vivo* using chimeric mice [39]. Prophylactic treatment with anti-CD81 antibodies completely protected uPA-SCID immunodeficient mice with humanized liver from a challenge with HCV consensus strains of different genotypes. The authors put forward the concept of CD81 as a clinical target for HCV prevention, especially in the context of liver transplantation (LT). Indeed, the saturation of the donor liver with anti-CD81 antibodies before and in the first days following graft reperfusion may prevent reinfection [38]. Recently monoclonal antibodies directed against claudin-1, another receptor of HCV, were developed [40]. They exhibited efficient inhibition against HCV infection in culture cells and primary hepatocytes, with an IC50 in the nanomolar range. These antibodies have only been evaluated *in vitro*, but in the perspective of a clinical use, they could prevent reinfection of the graft after LT. Furthermore, as claudin-1 is also implicated in cell-cell transmission [41], these antibodies might restrain virus spread in chronically infected patients.

The combination of two antibodies Ab^XTL^68 and Ab^XTL^65, which recognize different epitopes on HCV E2, is being evaluated in an ongoing study to assess the safety, tolerability and antiviral activity of increasing single and multiple doses in patients with chronic HCV infection [42,43] (Table 1). The combination of both antibodies, HCV-AB6865, was characterized biochemically *in vitro* and functionally *in vivo* using the HCV-Trimera mouse model (immunodeficient mouse transplanted with *ex vivo* HCV-infected human liver fragments). HCV-AB6865 was efficient at treating HCV infection of human liver fragments and at reducing the mean viral load in HCV-positive animals. It seemed more efficient than each agent taken separately and may diminish the risk of emergence of escape mutants.

The safety, pharmacokinetics and antiviral effect of Civacir, an immune globulin enriched in human hepatitis C polyclonal antibodies (HCIG), were studied in patients undergoing LT for chronic hepatitis C [42,44] (Table 1). A phase II “proof-of-concept” clinical trial was initiated in 2007, involving 20 patients receiving 400 mg/kg of Civacir. During this trial, the progression of liver fibrosis was studied on biopsies. Liver enzymes and HCV RNA levels were analyzed in liver and serum; safety and tolerance were evaluated. However no result has yet been made public.

A recent study revealed that mAbs directed against the domain III of DENV E protein potently neutralized infection in a mouse model of lethal dengue fever, illustrating the strong potential of an antibody-based therapy on severe forms of the illness [45]. 

### Other attachment inhibitors

#### Inhibitors targeting envelope proteins

Another approach to inhibit virus attachment is to use soluble proteins acting as molecular decoys. In the therapeutic arsenal against HIV, a recombinant soluble form of CD4 binding to gp120 was tested; however its activity in clinical trials was disappointing [37]. Pro-542, a tetravalent CD4-IgG2 fusion protein incorporating four copies of the Env-binding domains of CD4, was more promising (Table 1): reductions in plasma HIV-1 RNA levels were observed in phases I and II trials in patients with advanced HIV disease. Pro-542 bound HIV-1 gp120 with nanomolar affinity and neutralized HIV-1 regardless of genotype [46]. It was also well tolerated [11,37,46]. 

Small molecules such as azaindole derivatives also inhibited the interaction between HIV gp120 and CD4 [47,48]. One of those, BMS-378806, displayed a nanomolar IC50, an activity independent of the HIV coreceptor used and a cytotoxicity above 300 µM. Pharmacokinetic parameters of BMS-378806 were analyzed in rat, dog and monkey, resulting in a safe profile and attractive pharmaceutical properties. Using resistant viruses, Wang *et al.* identified mutations located in the pocket of gp120 that interacts with CD4, suggesting that the molecule could interfere with this interaction. Guo *et al.* confirmed this hypothesis and showed a direct interaction of BMS-378806 with gp120. Thus it could inhibit the binding of HIV-1 gp120 protein to CD4 *via* a specific and competitive mechanism. 

The EB peptide (for entry blocker) developed by Jones *et al.* against Influenza viruses, exhibits a broad-spectrum antiviral activity [49]. This 20-residue peptide does not induce significant cytotoxicity until 50 µM, and exhibits an IC50 in the low µM range. Furthermore, the protective activity of EB was demonstrated *in vivo* in mice. The authors suggest that EB may lead to a conformational change in HA, decreasing its affinity for sialic acid receptors on the cell surface. Alternatively, EB may interact with or near the receptors, blocking this site or causing steric hindrance during HA/receptor docking. EB may also induce aggregation of the virions, resulting in decreased cell binding. More recently, Rajik *et al.* described a smaller peptide (7 residues) which binds to HA and prevents cell attachment of the influenza virus [50]. This peptide possessed good antiviral activity *in vitro* and *in ovo*. 

In the case of HCV, various antiviral proteins and small molecules can be mentioned. The lectin cyanovirin-N (CV-N) is a protein isolated from an aqueous extract of the cyanobacterium *Nostoc ellipsosporum*. The authors demonstrated that CV-N inhibited cell entry at low nanomolar concentrations. CV-N targets the glycans of HCV envelope proteins and blocks the interaction between E2 and CD81, thereby preventing viral entry into target cells [51]. This protein had initially been discovered based on its potent activity against HIV. Indeed, Boyd *et al.* observed an anti-HIV activity with an IC50 in the nanomolar range. They measured a direct interaction between CV-N and HIV gp120, leading to viral membrane fusion inhibition [52]. Moreover, CV-N also inhibited several strains of influenza viruses [53]. Since various envelope proteins of viruses are glycosylated, such an approach could be promising for the development of viral entry inhibitors. Actinohivin (AH), a protein isolated from an *Actinomycetes* and binding high-manose type sugar chains of gp120, inhibited HIV entry [54,55,56,57,58]. Three segments of AH, and, within these segments, several residues are essential for this activity [59]. The cooperative involvement of each segment of AH increased the AH-gp120 interaction [60]. Interestingly, AH is endowed with broadly neutralizing activity against laboratory-adapted HIV strains and a variety of X4 and/or R5 HIV-1 clinical clade isolates. 

Other carbohydrate-binding agents, plant lectins and the antibiotic pradimicin A specifically inhibited HCV and HIV entry, with a submicromolar EC50 for plant lectins and micromolar EC50 for pradimicin A [61]. Along similar lines, soluble chondroitin sulphate E (CSE) was shown to inhibit DENV infection as well as virus binding to host cells through interaction with the E protein [62].

#### Inhibitors targeting host receptors

Recently EWI-2wint, a cellular partner of CD81 within the tetraspanin web expressed in several cell types but not in hepatocytes, was described to modulate HCV cell entry [63,64]. Indeed, ectopic expression of EWI-2wint in an HCV-permissive hepatoma cell line inhibited viral entry by blocking HCV binding to CD81, most likely through interactions with CD81. However the molecular mechanism of EWI-2wint inhibition of HCV entry is still unknown. EWI-2wint may reduce CD81 accessibility to HCV E2 by steric hindrance, or the association of EWI-2wint with CD81 may induce conformational rearrangements in CD81, rendering it less accessible for HCV E2 interaction. Alternatively, EWI-2wint may interfere with actin polymerization during viral entry or block signaling pathways necessary for viral entry. EWI-2wint is not applicable in therapy since it is a full-length protein, but could be used as a template for the design of smaller molecules mimicking its antiviral activity. 

Molecules such as terfenadine and derivatives could inhibit HCV E2/CD81 interaction [65]. These cyclic molecules were developed to interact with the large extracellular loop (LEL) of CD81, to compete with HCV binding. Terfenadine displayed moderate HCV inhibition, with only 27% of inhibition of CD81/E2 interaction at 50 µM, and its derivatives inhibited up to 69% of CD81/E2 interaction at the same concentration. These molecules, tested at concentrations ranging from 0.5 to 5 µM in an HCVcc infectivity assay, displayed a good inhibitory effect. Since similar inhibition was obtained with compounds reducing infectivity but devoid of any activity in a protein/protein interaction assay, the authors speculated that these molecules could interact with an additional target involved in viral infection.

## Viral Envelope Proteins as Targets for Entry Inhibitors

Developing antiviral molecules directed against viral envelope proteins is based on the critical role these proteins play in viral entry. Furthermore, molecules that do not act on cellular factors may display moderate to low toxicity. Conversely this type of inhibitors could induce viral resistance due to selection pressure on the virus leading to mutations in viral protein sequences. These molecules stabilize viral envelope proteins or alter their conformation, thereby blocking their oligomerization or the conformational changes required for fusion. 

This type of strategy was applied to RSV entry inhibition. Two small benzimidazole derivatives were developed, that interacted with the RSV F fusion protein : BMS-433771 [66,67] and TMC353121 [8]. BMS-433771 is an orally active drug with an efficacy in the nanomolar range. Cianci *et al*. used a photoaffinity analogue of BMS-433771 in order to directly probe the site of interaction of the inhibitor with the fusion protein. A tyrosine residue was mapped within this hydrophobic cavity. Thus BMS-433771 could block interaction between the two heptad repeats and could thus interfere with the formation or consolidation of key structures within the hairpin, essential for membrane fusion. As class I fusion proteins share this common mechanism of action, the development of such a molecule is very promising. TMC353121 acted through a similar mechanism but could inhibit fusion by causing a local perturbation of the native 6HB conformation, rather than by blocking the formation of the 6HB [8].

Influenza virus A/H3N2 is sensitive to a class of *N*-(1-thia-4-azaspiro [4,5] decan-4-yl) carboxamide molecules, acting with an IC_50_ in the µM range and a cytotoxicity above 100 µM [68]. However, these molecules are not efficient against other influenza viruses such as H1N1, H5N1, H7N2 or B viruses. The authors reported that the compound named 4c, one of the most potent molecules in this class, inhibited HA-mediated membrane fusion by preventing HA conformational changes at low pH. By *in silico* docking approaches, they predicted the position of a potential 4c binding pocket in the HA trimer, involving arginine and glutamic acid residues. These molecules are very useful laboratory tools for the design of compounds with improved antiviral effect and a broader spectrum. In the same way, Russel *et al.* identified a hydrophobic pocket between HA monomers as a potential target for antiviral drugs [69]. Indeed, an inhibitor of membrane fusion (*tert*-butylhydroquinone) was shown to occupy this site and to stabilize the neutral pH structure through intersubunit and intrasubunit interactions, presumably inhibiting the conformational rearrangements required for membrane fusion. The discovery of this pocket as an inhibitory site could lead to the development of potential anti-influenza drugs.

Cyclic molecules targeting the β-OG pocket of the Dengue E fusion protein, described by Harrison and coworkers and located nearby the flexible hinge region (Figure 4), could inhibit virus entry. Indeed, compounds targeting this hydrophobic pocket are thought to interfere with conformational changes in the envelope protein that are essential for fusion [20]. Several teams, using *in silico* screening, discovered inhibitors targeting this pocket, active against DENV, YFV, WNV and JEV [19,70,71,72]. Immunofluorescence studies indicated that in the presence of one of these compounds, DENV was blocked in the endosomes after receptor-mediated endocytosis [71]. This was not due to alteration of the endosomal pH, but the precise mode of action remains to be determined. The authors showed that this molecule could specifically bind to DENV, and computational chemistry revealed the position of the compound deeply buried in the β-OG pocket. Therefore the pocket in E protein could be a promising target for antivirals.

The E protein of flaviviruses could also be the target of antiviral peptides or protein stemming from the protein itself [73,74]. Hrobowski *et al.* identified peptides derived from the E protein, inhibiting WNV and DENV infectivity [73]. DN59 (33 residues), corresponding to the stem domain of DENV, inhibited DENV and WNV entry. WN83 (25 residues), corresponding to WNV E domain IIb, inhibited WNV but not DENV infectivity. The mode of action of these peptides appeared to be sequence-specific, but the exact mechanism remains unclear. They may interfere with the interactions between the stem and other portions of the viral fusion proteins, but inhibition through interference with some target cell surface components cannot be ruled out. However, the domains encompassing these peptides, IIb and the pre-anchor stem, are likely involved in structural rearrangements within the fusion protein during the fusion process, rather than in direct interactions with cellular receptors. Such studies could lead to the development of peptidomimetic molecules that would use the same mode of action. More recently, Schmidt *et al.* identified peptides derived from the stem region of Dengue E [74]. These peptides bind to the trimeric postfusion conformer but not to the prefusion dimer, and inhibit infectivity by blocking viral fusion.

Recombinant proteins corresponding to domain III of either the alphavirus E1 protein or the flavivirus E protein inhibited alpha- or flavi-virus infection [75,76]. These proteins could interfere with protein rearrangement during fusion [75] or could block virus attachment [76]. Although they are unlikely to be used as antivirals as such, their strong inhibitory activity has important implications for the development of clinically useful inhibitors of the fusion reaction brought about by class II proteins.

Some classes of molecules can act by direct binding to the viral envelope protein. Amphipathic polymers of DNA such as phosphorothioate oligonucleotides (PS-ONs) have recently emerged as an attractive option. In 2006, Vaillant *et al.* analyzed their activity on HIV infection [77]. The polymer inhibitory activity was dependent on its amphipathic properties and size, but was sequence-independent. Importantly, the phosphorothioate group plays an essential role by increasing the overall hydrophobicity. PS-ONs blocked entry of both CXCR4- or CCR5-tropic HIV-1 in a cell fusion assay, with an IC50 lower than 300 nM. Due to their hydrophobicity, PS-ONs could interact non specifically with hydrophobic molecules such as serum proteins, which could affect their *in vivo* efficacy. The activity of these PS-ON against HCV infection was also tested in 2009 by Matsumura *et al*. [78,79]. PS-ONs were shown to potently inhibit HCV infection at submicromolar concentrations and were similarly active against HCV pseudoparticles of various genotypes. PS-ONs also inhibited HCV infection *in vivo,* in a chimeric mouse infected with HCV from patient’s serum [78]. However, since HCV fusion is most likely achieved by a class II fusion protein, the mechanism of action against HIV and HCV may not be conserved, and it remains to be determined in HCV infection. A clinical evaluation started in 2007 to test a PS-ON (REP 9AC) in HCV/HBV co-infected patients (Table 1), is currently performed in Southeast Asia to evaluate its safety and antiviral efficacy (http://www.girindusglos.com/resources/GLOS2009Day1/REPLICorPresentationGLOS2009ver2public.pdf).

In the case of HCV, the development of molecules targeting a specific conformation or region of one of the envelope proteins is severely hampered by the lack of 3D structure of these proteins and the lack of HCV models until recently [23,24]. Recently, EI-1 has been identified from a library of small molecules [80]. It is active against genotype 1a and 1b HCV, with median EC50 values of 134 and 27 nM respectively against infection. EI-1 inhibited both cell-free and cell-to-cell entry. Experiments with resistant viruses suggested that EI-1 could act on the E2 envelope glycoprotein.

## Blocking Cellular Proteins to Inhibit Virus Entry

Targeting cellular receptors could constitute a good strategy since it could limit mutational resistance and reduce viral escape. Nevertheless, this strategy may be delicate because the targeted host proteins play cellular roles.

For HIV, several molecules targeting cellular receptors are currently on clinical trials or are already used in clinics. For example, maraviroc and vicriviroc are two small molecules, antagonists of CCR5 [11,12,37]. The result of two phase III clinical trials studying vicriviroc indicated its lack of efficacy, leading Merck to terminate its development programme in July 2010 (http://anewmerckreviewed. wordpress.com/2010/07/15/merck-makes-it-official-sgps-vicriviroc-hiv-rd-program-terminated/). CCR5 is a membrane protein with seven transmembrane domains (TMD); maraviroc was shown to interact with a tyrosine residue in the third TMD and a glutamate residue in the seventh TMD of CCR5. The transmembrane pocket is nearby but distinct from the extracellular domains of CCR5 recognized by gp120. Maraviroc therefore alters the conformation of specific locations of the CCR5 ectodomain where HIV binds (Table 1). *In vitro* studies showed that it was able to inhibit entry of a panel of clinical HIV isolates, with an IC_50_ in the nanomolar range. However, since it targets CCR5, it cannot inhibit the entry of HIV strains using CXCR4. Maraviroc was developed by Pfizer Sandwich Laboratories and is now in clinical use for the *per os* treatment of HIV-1 infection. Pharmacokinetic studies demonstrated good oral bioavailability, safety and tolerability. Although the efficacy was globally good, some virological failures were observed. However, viruses highly resistant to maraviroc remained sensitive to other CCR5 antagonists. This could be due to the fact that each antagonist would not occupy exactly the same site, selecting distinct conformations of the bound receptor. Other CCR5 antagonists were developed and studied, such as aplaviroc (discontinued after reports of hepatotoxicity), PF-232,798 or INCB009471 [12,37] (Table 1). A few antagonists of CXCR4 were studied such as AMD3100 which is a potent antiviral *in vitro*, but development programs have been terminated. Indeed, patients had significant levels of circulating virus using CCR5, therefore not inhibited by AMD3100. This might argue for the co-administration of a combination of antagonists against CCR5 and CXCR4. Another strategy to interfere with HIV receptor(s) could be the down-regulation of CCR5 expression. Rapamycin is a macrolide antibiotic already in clinical use for the prophylactic treatment of renal graft rejection. This drug decreases CCR5 expression in normal T cells and macrophages, and inhibits the *in vitro* replication of HIV-1 strains using CCR5 [81,82]. Gilliam *et al.* tested this molecule on CCR5 expression in macaques. The regimen was well tolerated and the authors observed a reduction in the levels of CCR5 mRNA. Nicoletti *et al.* analyzed the effect of rapamycin on chimeric mouse infected with HIV using CCR5 [82]. As expected, they observed a decrease in HIV RNA levels in the blood of treated mice. Although these data suggest that rapamycin would mainly act on HIV infection by inhibiting CCR5 expression, it is possible that other effects as well might contribute to its antiviral activity. It might be used in combination with a CCR5 antagonist to reduce the doses of the latter and increase antiviral activity. 

Along the same lines, two inhibitors against the influenza virus were developed, based on the concept of decreased receptor (sialic acids) accessibility. Among them, clarithromycin, a macrolide antibiotic, used to treat pharyngitis and tonsillitis, could inhibit two steps of the influenza virus entry process (Table 1). Clarithromycin reduces the expression of sialic acid residues on the surface of airway epithelial cells, thereby lowering the virus binding capacity. It was also shown to decrease the number of acidic endosomes in the cell, inhibiting endosomal escape [1,83]. Clarithromycin decreased viral titers and RNA of influenza virus in a concentration-dependent way, with an efficacy in the micromolar range and with a maximal inhibitory effect obtained at 100 µM. 

The other inhibitor is DAS181 (namely Fludase) (Table 1), also reducing the presence of sialic acids at the cell surface. It is a recombinant fusion protein composed of the sialidase (neuraminidase) catalytic domain derived from *Actinomyces viscosus*, fused with a cell-surface-anchoring sequence [84]. DAS181 showed potent antiviral and cell-protective efficacies against a panel of laboratory strains and clinical isolates of influenza A and B viruses, with an inhibition of virus replication lower than nanomolar. DAS181 was also efficient at preventing and treating influenza infection in mice. DAS181 is administered by aerosol to remove sialic acids from the airway epithelium. In their study, Malakhov *et al.* demonstrated that DAS181 cleaved sialic acids used by both human and avian influenza viruses, and indicated that 50 to 70% cell surface sialic acid removal afforded >90% cell protection against influenza virus. Furthermore, DAS181 showed *in vitro* efficacy against clinical isolates resistant to neuraminidase inhibitors such as oseltamivir [85]. Thus, DAS181 may offer promising therapeutic options against seasonal or pandemic influenza viruses resistant to currently available antiviral drugs. At present a phase II clinical trial is recruiting subjects with influenza-like illness to test safety and efficacy of DAS181.

For HCV, two inhibitor molecules (ITX5061 and ITX4520) that target the SR-BI receptor have been developed and are currently in clinical trials (Table 1). They are both orally bioavailable. ITX5061 inhibits infection by HCV of genotypes 1 and 2, with an EC_50_ around 0.1 nM [42,86]. ITX4520 is currently in phase I clinical studies [42]. Antiviral strategies targeting HCV receptors are still limited. New HCV receptors were recently discovered (claudin-1 [25] and Occludin [26]), but their role and interaction with HCV are not well characterized yet.

Another strategy is to target host proteases involved in the maturation of viral envelope proteins. Thus, a small oxocarbazate molecule inhibiting human cathepsin L inhibited cell entry of the SARS (Severe Acute Respiratory Syndrome) coronavirus and of Ebola pseudotyped virus, with nM EC50 [87]. Processing of the glycoprotein precursor (GPC) of arenaviruses by the cellular pro-protein convertase site 1 protease (S1P) is crucial for cell-to-cell propagation of infection [88]. Thus, Rojek *et al.* developed peptide-based S1P inhibitor (dec-RRLL-CMK) that blocked cell-to-cell propagation of infection of lymphocytic choriomeningitis virus (LCMV). Concerning Influenza virus, post-translational proteolytic cleavage of HA0 into HA1 and HA2 is a prerequisite for viral entry into host cells and membrane fusion activity [89,90]. At least seven different cellular trypsin-type proteases such as HAT (Human airway trypsin-like protease) could be involved in the cleavage, suggesting that strategies targeting these proteases could therefore be of great anti-flu potential.

## Target: Membranes and Proteins

Membranes and their lipid composition play an essential role in viral entry and in particular at the fusion step. Therefore targeting virus and/or cell membranes could reveal promising. Samaritan Pharmaceuticals Inc. has discovered that SP01A, a molecule of unknown structure due to patent protection (Table 1), reduces intracellular cholesterol and blocks the organization of lipid rafts in the cellular membrane, ultimately preventing HIV fusion (http://www.samaritanpharma.com/aids_hiv_program_sp-01a.asp and http://www.clinicaltrials.gov/ct2/results?term=SP01A). SP01A significantly lowered the amount of HIV in blood, had a favorable safety profile with minimal side effects, and was well tolerated. Moreover, cell culture assays demonstrated that SP01A had comparable or greater efficacy than currently approved anti-HIV drugs in preventing HIV virus replication, with a low toxicity toward human cultured cells. In 2005 and 2006 two randomized phase II double-blind clinical trials aimed at testing safety and efficacy of SP01A administered orally as a monotherapy treatment in HIV-infected patients (http://clinicaltrials.gov, identifiers: NCT00299897 and NCT00113412).

In 2008, using a peptide library, Cheng *et al.* found a novel broad-spectrum inhibitor of viral entry [91]. This peptide (C5A) is composed of residues 3–20 of the α-helical N-terminal membrane anchor domain of the HCV NS5A protein [92,93]. It inhibited entry of several viruses, including HCV, WNV, DENV and HIV, but was inefficient on Influenza A virus or HBV [91]. The activity of this peptide has also been studied in the context of HIV infection [94]. A direct virocidal effect was observed at submicromolar concentrations *in vitro*, suggesting that it could permeabilize the viral phospholipid bilayer. Interestingly, this peptide seems to merely act on the viral envelope, and not on cell membranes. C5A prevents and suppresses HCV infection *in vitro,* and displays a favorable profile *in vivo*, with a low toxicity when administered intravenously in mice. The amphipathic α-helical structure is necessary but not sufficient for its virocidal activity. This activity further depends on C5A amino acid composition but not primary sequence. However, due to its propensity to interact with lipids (membranes), it may interact with several hydrophobic molecules, thereby interfering with host metabolism. Furthermore, peptides must be administered intravenously, which might be difficult to bear for patients and which could limit the bioavailability due to interactions with serum proteins.

Like all fusing bilayers, viral envelopes locally adopt hourglass-shaped stalks during the initial stages of fusion, a process that requires local negative membrane curvature. St Vincent *et al.*, identified a family of synthetic rigid amphiphiles, RAFIs (rigid amphipathic fusion inhibitors, nucleosides derivatives), that inhibit the infectivity of HCV and Herpes Simplex virus (HSV), by interfering with the negative curvature required for initial stages of fusion [95]. Similarly Wolf *et al.* identified an antiviral small molecule (LJ001) active against Influenza A, HIV-1 and flaviviruses [96]. LJ001 specifically intercalated into viral membranes, irreversibly inactivated virions while leaving functionally intact envelope proteins, and inhibited viral entry at a step after virus binding but before virus-cell fusion.

Membranes can be considered as concentrators of small molecules with pharmacological activity, and in the case of antivirals this concentration could potentiate their antiviral properties.

It is established that Enfuvirtide (dp 178; T20; Fuzeon™), a peptide fusion inhibitor used against HIV-1, acts in this way [11,37] (Table 1). Enfuvirtide (Enf) was the first fusion inhibitor approved for clinical use in HIV infection and became available in 2003. This 36-amino acid peptide corresponds to the amino acid residues 127–162 of the viral glycoprotein gp41. Enf binds, at an intermediate stage of fusion, to the first heptad-repeat (HR1 - aa 36–45) of gp41 and blocks its interaction with HR2 by competitively binding to the hydrophobic grooves of the HR1 trimer. This blocks the formation of the “hairpin” structure and the subsequent fusion reaction. Several studies also demonstrated that the inhibitory effect of Enf could be explained by membrane interactions. Indeed, the presence of three non consecutive tryptophans, one tyrosine and one phenylalanine in Enf sequence suggests that it could interact interfacially with membranes. Enf was indeed shown to bind to, concentrate and oligomerize within the membrane interface [97,98,99]. Cell membranes can therefore play a role of reservoir for Enf, locally concentrating the molecule. Thus, the membrane can act as a “catalyst” to the binding reaction between gp41 and the peptide. Since the mechanism of action of Enf is different from other classes of anti-HIV medication, it is effective in patients who have failed other therapies due to emergence of resistant virus. In therapeutic use, Enf is associated with other anti-HIV agents and is administered by subcutaneous injection twice daily at a dose of 90 mg. The drug has minimal systemic toxicity, but the frequent occurrence of painful injection site reactions has limited long-term use. 

Recently, a peptide inhibitor of HIV tagged with a cholesterol molecule (C34-Chol) was developed by Ingallinella *et al.,* in order to increase peptide's efficacy by enhancing its membrane association [100]. C34 and Enf share a common sequence of 24 residues [97]. Without its cholesterol-tag, C34 inhibits HIV entry by binding to the prehairpin intermediate and preventing its transition to the 6HB structure. C34-Chol showed dramatically increased antiviral potency, with an IC50 value 50-fold lower than C34 (4 pM vs 250 pM, respectively). The addition of the cholesterol group could therefore create a specific enrichment of the inhibitor peptide in lipid microdomains, where HIV-cell fusion occurs. Moreover, derivatization with cholesterol extends the half-life of the peptide *in vivo*.

Peptides derived from the HR2 region of the F protein of paramyxoviruses inhibited the infectivity of homologous viruses. Porotto *et al.* demonstrated that the addition of a cholesterol tag onto such a peptide can increase its antiviral potency by two logs [9]. Indeed its IC_50_ was ~ 7 nM, whereas the IC50 was approximately 100-fold higher for the untagged version of the peptide. Thus, the authors suggested that the cholesterol tag helped increase the rate of inhibitor association with the pre-hairpin intermediate, *via* its pre-concentration in the membrane. 

In the HBV life cycle, during the synthesis of the envelope protein L, the pre-S1 domain is myristoylated and translocated through the endoplasmic reticulum (ER), and this modification of pre-S1 is essential for infectivity [101]. Interestingly, a peptide derived from the pre-S1 domain of the L envelope protein of HBV, exhibits a stronger antiviral activity when the peptide is tagged with a myristoyl moiety [102]. Preincubation of primary tupaia hepatocytes (used as a model for HBV infection [102]), with the myristoyl-tagged version of pre-S1 (aa 2-48) completely inhibited HBV infection at 10 nM, whereas more than 10 µM of the untagged version of pre-S1 are necessary for a complete infection inhibition. Interestingly, the replacement of myristic acid by other fatty acids resulted in changes in the inhibitory activity, most notably by a decrease in the IC_50_ to picomolar concentrations for longer unbranched fatty acids or stearic acid [101,103]. In the presence of this peptide, viral particles did not bind to tupaia hepatocytes. This could suggest a specific binding of the peptide to a cellular receptor necessary for HBV attachment to hepatocytes, although an additional interaction with a viral protein cannot be excluded and may contribute to the inhibitory effect. The interaction of the lipopeptide with this putative receptor molecule was probably enhanced by the anchorage of the hydrophobic myristoyl moiety into the membrane. In the near future, HBV preS-derived lipopeptides could be administered to prevent HBV infection, such as in post-exposure prophylaxis or to avoid reinfection of the liver graft after transplantation of HBV-infected individuals. Further studies are needed to evaluate if this inhibitor may play a beneficial role in chronically-infected patients.

Lipophilic drugs targeting cellular receptors have also been developed against Influenza virus infection. Prior to membrane fusion, the low pH into the endosome activates the influenza virus M2 proton channel to conduct protons across the viral envelope. This results in the acidification of the viral particle lumen, and promotes virus uncoating within endosomes [18,104]. This M2 channel is the target of lipophilic adamantanes (tricyclodecane derivatives), such as amantadine and rimantadine which differ by an ethylamine moiety in position 1 (Table 1). Amantadine was one of the most studied inhibitors of the influenza A virus M2 proton channel [104,105]. In spite of severe side effects, amantadine is used as an anti-influenza drug in clinics since 1966, and also in Parkinson’s disease [106]. Since then, the derived compound rimantadine has been approved as well. Amantadine can reach the channel after partitioning into the membrane [107]; indeed several experiments suggest that amantadine is significantly more soluble in lipid bilayers than in aqueous solution and adopts a preferential interfacial location. Unfortunately, the rate of onset of virus resistance to these drugs is globally increasing and overlapping cross-resistance between amantadine and rimantadine is observed [108,109]. Monitoring the emergence and spread of resistant viruses is therefore urgently needed in order to apply appropriate treatments to patients.

Arbidol (Arb), a broad-spectrum antiviral which demonstrated activity against a number of enveloped and non-enveloped viruses, was initially used for prophylaxis and treatment of infections by influenza A and B viruses [110,111,112]. 

Arb is a small indole-derivatives molecule that was first marketed in Russia in 1993 and in China in 2006 [113]. It is generally administered orally at 200 mg 4 times a day for 3–5 days against flu, and it is given in 200 mg doses once a day for 5–10 days for prophylaxis of subjects in contact with symptomatic patients [110]. This treatment was shown to reduce the duration of illness by 1.7–2.6 days in infected patients, and to prevent the development of post-influenza complications. Furthermore, the therapeutic efficacy of Arb was most pronounced when the drug was administered early in the infection. Although Arb is in clinical use for more than 15 years, no Arb-resistant viruses have been isolated so far, a major advantage of Arb over adamantanes [110,111,113]. Clinical trials conducted in more than 10,000 patients and more than 15 years of experience with Arb in Russia revealed that Arb is well tolerated with no or minor side effects. Arb is an entry inhibitor and more precisely a fusion inhibitor. Mechanistically, Arb inhibits influenza virus-induced membrane fusion, and immuno-modulatory effects were reported. Arb can interact with influenza HA, stabilize it and prevent low pH-induced conformational changes leading to membrane fusion. This stabilization consequently leads to the inhibition of endosomal membrane fusion [111].

Recently, an antiviral action against HCV was demonstrated on acute and chronic HCV infection [114,115,116]. Arb inhibits HCV membrane fusion and cell infectivity of various genotypes with an IC50 about 10 µM [117]. Pécheur *et al.* have already shown that Arb could interact with micelles and membranes of various compositions [114]. In our recent work, Arb location in the membrane was studied, and Arb was shown to mainly interact with the polar head-group of phospholipids at the membrane interface, with an apparent affinity in the low micromolar range [117]. This indicates that at least part of Arb inhibitory activity could be explained by its membranotropism. Its interaction with phospholipids would locally perturb membrane fluidity, a key parameter to the fusion process, thereby rendering the lipid bilayer less prone to fusion. Moreover its indole structure allows an interaction with aromatic residues such as tryptophan. Indeed, we showed a direct interaction between Arb and tryptophan residues of various membranotropic peptides in membrane environments, with an apparent affinity in the µM range. Interestingly, apparent binding affinities to lipids and tryptophan residues are in the same range as the IC_50_ of HCV membrane fusion (7–10 µM). Since tryptophan residues of membrane proteins are known to bind preferentially at the membrane interface, these data suggest that Arb could increase the strength of HCV glycoprotein's interactions with the membrane, due to a dual binding mode involving aromatic residues and phospholipids. The resulting complexation would reduce the overall speed of the fusion reaction rather than block a specific protein conformation, leading to inhibition of the expected HCV glycoprotein conformational changes required during the membrane fusion process. 

Arb safety demonstrated against influenza infection, together with its *in vitro* efficacy against HCV suggest that Arb could be a good anti-HCV drug candidate when co-administrated with other antiviral molecules during HCV treatment. It could help improve the efficacy and/or reduce the doses of the current treatment.

## HCV Inhibitors Targeting Unknown Targets

In this part we will detail antiviral agents directed against HCV entry, for which the precise target is unknown. A 16-residue polypeptide, containing a portion of the E2 transmembrane domain, was shown to inhibit cell entry of HCV pseudoparticles [118]. Its IC50 was approximately 300 nM, and it had no effect on VSV nor HIV pseudoparticles entry, suggesting a specific action against HCV. Its antiviral activity was dependent upon the L-configuration of the amino acids and the overall hydrophobic character of the peptide. But the hydrophobic character was not sufficient since the native sequence was required for optimal activity. This peptide did not exhibit direct virocidal activity against HCV pseudoparticles, but acted at a post-binding step. 

Several plant extracts were recently shown to block HCV entry. The antiviral iridoid isomers lamiridosins A and B, extracted from the flowering tops of *Lamium album*, inhibit HCV entry *in vitro* with an IC_50_ in the µM range [119]. Since they were not active against VSV entry, the authors suggested that these drugs could be selective to HCV. Their exact mechanism of action has not been identified yet, but at least interference in the CD81/E2 interaction could be ruled out.

Silymarin (SM) is another plant extract obtained from the seeds of milk thistle from *Silybum marianum* [120] (Table 1). The extract contains multiple flavonolignans including silybin (a racemic mixture of diastereoisomers A and B), isosilybins A and B, silychristin, silydianin and other phenol compounds. SM is used for centuries against hepatic disorders, and its hepatoprotective role is known for hundreds of years. It is thought to have antioxidant, anti-inflammatory, immunomodulatory and antitumor effects. Moreover, SM did not display major side effects [121,122,123]. Currently, the milk thistle extract is marketed worldwide including the United States as capsule/tablet of silymarin and silibinin with enhanced bioavailability under various trade names, such as Siliphos^®^, Silipide^®^ and Legalon^®^. Surveys indicated that persons with chronic hepatitis C (CHC) often use herbals, especially SM, with the hope to improve the modest response to certain antiviral therapies and to reduce side effects [124]. The use and potential effects of SM was studied in a trial involving patients with advanced CHC and non-responders to prior therapies. This study concluded that users of SM had similar alanine aminotransferase and HCV levels than those of non-users, but exhibited fewer symptoms and a somewhat better quality of life. Clinical trials to test the efficacy and safety of SM are programmed and currently recruiting HCV-infected patients (http://clinicaltrials.gov, identifiers: NCT00680342, NCT01018615 and NCT00755950). A clinical trial studied the safety and efficacy of silibinin (the major component of silymarin) in HCV-infected patients non-responders to pegylated-IFN/ribavirin (PegIFN/RBV) [125]. In this study, a substantial antiviral effect against HCV in non-responders was observed, with a dose-dependent reduction of viral load after 1 week of combined silibinin and PegIFN/RBV therapy. Recently a successful suppression of early HCV reinfection after liver transplantation with a 14-day course of silibinin monotherapy was reported [126]. Several articles recently showed an action of SM and its components at various stages of HCV infection [123,127,128]. However, the mechanisms of action remain unclear. SM and its different components exhibited an inhibition of HCVcc infection with an IC_50_ of 6 to 245 µM. We observed an inhibition of HCV entry by SM with no interference with virus/cell binding, suggesting that the inhibition could occur at a post-binding stage. Using a lipid mixing fusion assay, we demonstrated that SM inhibited membrane fusion up to 90 % at about 20 µM, with an IC_50_ around 5 µM, thus reinforcing the notion that SM could inhibit HCV entry at the fusion stage [128]. In a recent study we compared the antiviral effect of silibinin (insoluble in aqueous solution, orally administered) with that of its intravenous formulation Legalon-SIL (Wagoner *et al.*, under revision). Legalon-SIL differs from silibinin by two succinate moieties covalently attached to each diastereoisomer. Interestingly Legalon-SIL displayed a much more potent inhibitory effect on HCV fusion than silibinin (IC_50_ 11 μM *vs* ca. 50 μM, respectively). We therefore suggested that, due to their phenylbenzopyrone chemical structure and thereby hydrophobicity, SM and its components could act by incorporating into lipid membranes. This could lead to stabilization of membranes which would in turn become less prone to fusion.

## Conclusions

In spite of resistance issues, the successful development and regulatory approval of enfuvirtide, maraviroc or amantadine have shown that viral entry is a valid target for therapeutic intervention. It seems obvious that in the near future effective treatments against HCV and other enveloped viruses will become available. Possibilities include optimized small molecule antiviral agents, peptide or protein and nucleic acid-based therapies. These inhibitors target various steps of virus cell entry and both viral and cellular components, since host and viral targets have been proven valid for antiviral development. Interestingly, inhibitors that concentrate into/onto the membrane in order to target a protein involved in the entry process, such as arbidol or HIV peptide inhibitors, could allow the use of doses compatible with therapeutic requirements. More generally, the discovery of molecules blocking several steps of the viral life cycle could lead to combination therapies that also permit to lower the respective dose of each molecule, and consequently the resistance and side effect issues. 
